# Supplementary material for: Intranasal Delivery of Lithium Salt Suppresses Inflammatory Pyroptosis in the Brain and Ameliorates Memory Loss and Depression-like Behavior in 5XFAD Mice
Source: J Neuroimmune Pharmacol. 2025 Mar 17;20(1):26. doi: 10.1007/s11481-025-10185-7 (PMC11914297; doi:10.1007/s11481-025-10185-7)
Supplement: Supplementary file 1 — Supplementary file1 (DOCX 158 KB) [file 11481_2025_10185_MOESM1_ESM.docx]

**Supplemental Figure and Legends**

**
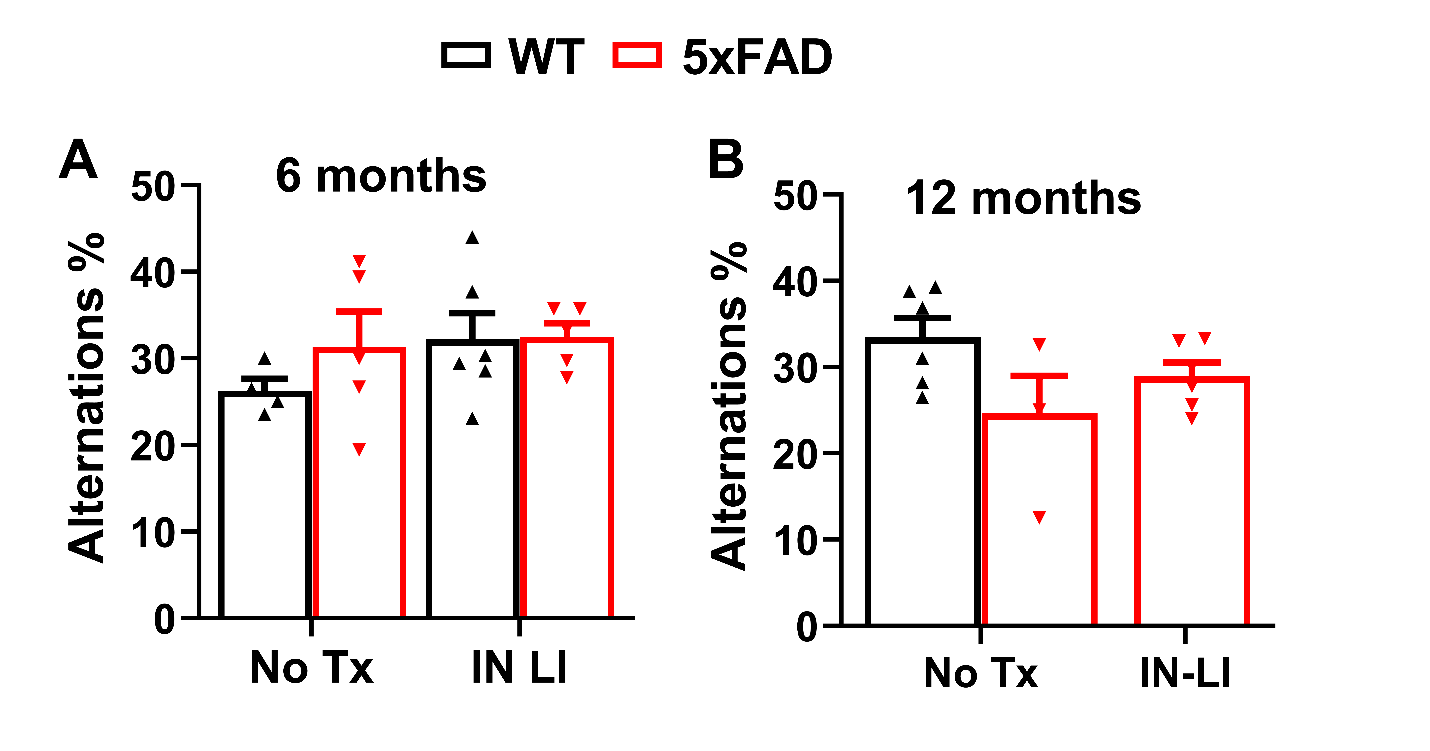
**

**Supplemental Figure 1. Effects of intranasal LiCl in RFV on cognitive function assessed by Y-maze test in 5xFAD mice.** Wild type (WT) or 5xFAD mice at 2 or 9 months of age received either no treatment (No Tx) or intranasal LiCl (IN LI, 3 mmol/kg) dissolved in RFV daily, Monday to Friday, for 12 consecutive weeks. Y-maze tests were performed at 6 (**A**) and 12 months of age (**B**), after completion of treatments. Data represents Means±SD from 4-6 mice (N=4-6) and was analyzed using the 2-way ANOVA followed by Tukey’s multiple comparison test.


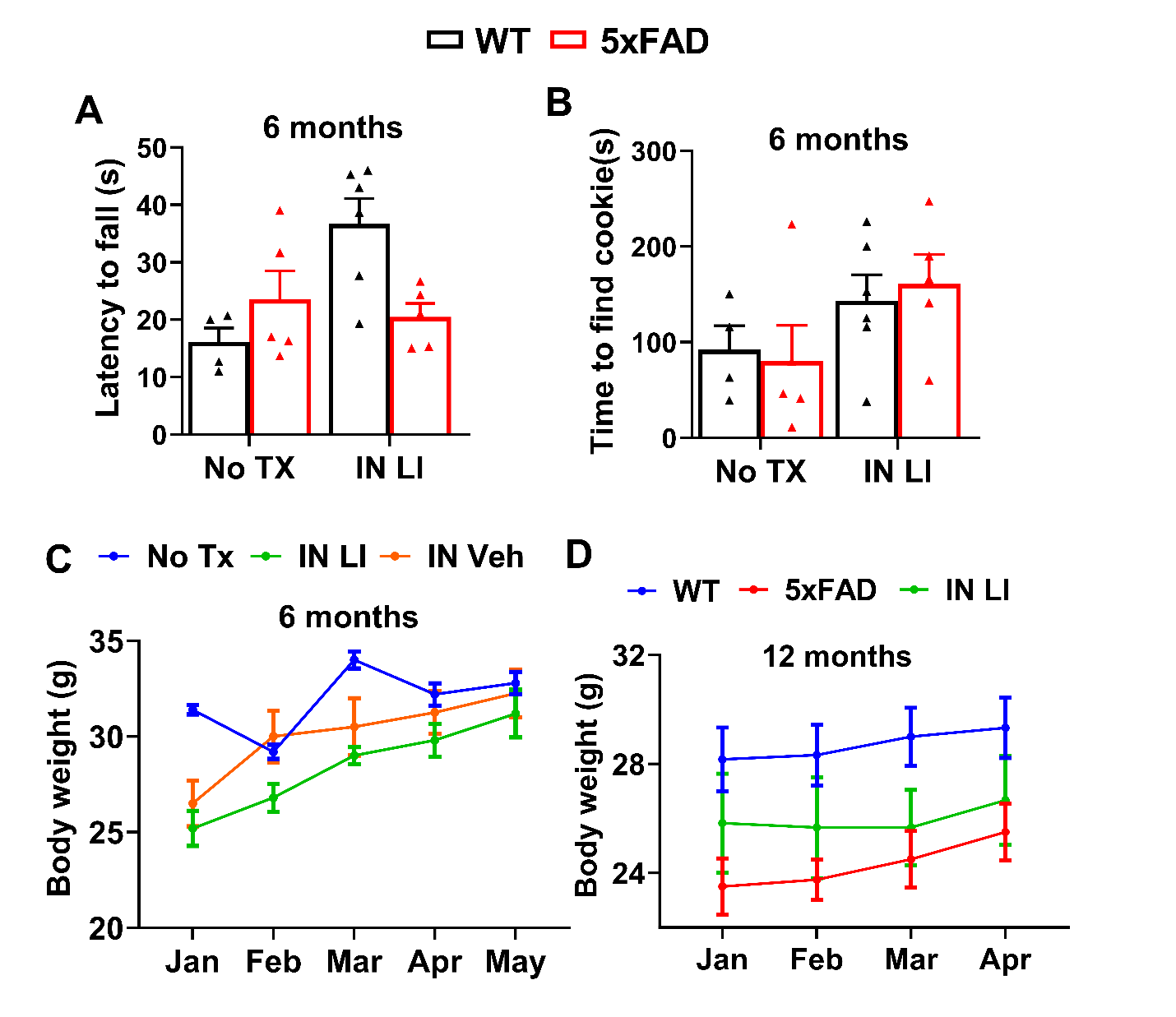


**Supplemental Figure 2: Effects of intranasal LiCl in RFV on motor and smell functions and body weight.** Wild type (WT) or 5xFAD mice received either no treatment (No Tx) or intranasal LiCl in RFV (INLI, 3 mmol/kg) for 12 consecutive weeks from 2 to 5 months of age. Motor (**A**) and olfactory (**B**) functions were determined by rotarod test and buried food test, respectively at 6 months of age. Body weights were determined monthly from the initiation of lithium treatment at 2 months of age **(C)** until the end of all behavioral tests at 12 months of age(**D**). Data are Means±SEM from 4-6 mice in each group.
